# Pattern Motion Processing by MT Neurons

**DOI:** 10.3389/fncir.2019.00043

**Published:** 2019-06-21

**Authors:** Parvin Zarei Eskikand, Tatiana Kameneva, Anthony N. Burkitt, David B. Grayden, Michael R. Ibbotson

**Affiliations:** ^1^NeuroEngineering Laboratory, Department of Biomedical Engineering, The University of Melbourne, Parkville, VIC, Australia; ^2^Faculty of Science, Engineering and Technology, Swinburne University of Technology, Hawthorn, VIC, Australia; ^3^National Vision Research Institute, Australian College of Optometry, Carlton, VIC, Australia

**Keywords:** computational modeling, middle temporal area, motion perception, pattern selectivity, vision

## Abstract

Based on stimulation with plaid patterns, neurons in the Middle Temporal (MT) area of primate visual cortex are divided into two types: pattern and component cells. The prevailing theory suggests that pattern selectivity results from the summation of the outputs of component cells as part of a hierarchical visual pathway. We present a computational model of the visual pathway from primary visual cortex (V1) to MT that suggests an alternate model where the progression from component to pattern selectivity is not required. Using standard orientation-selective V1 cells, end-stopped V1 cells, and V1 cells with extra-classical receptive fields (RFs) as inputs to MT, the model shows that the degree of pattern or component selectivity in MT could arise from the relative strengths of the three V1 input types. Dominance of end-stopped V1 neurons in the model leads to pattern selectivity in MT, while dominance of V1 cells with extra-classical RFs result in component selectivity. This model may assist in designing experiments to further understand motion processing mechanisms in primate MT.

## Introduction

The middle temporal area (MT or V5) within the extrastriate primate visual cortex contains a high proportion of direction-selective neurons (Dubner and Zeki, 1971; Born and Bradley, 2005). MT is regarded as the gateway to the perception of motion because it is the first area largely dedicated to motion processing in the cortical visual pathway and direct electrical stimulation of MT alters the direction of visual motion perception in monkeys (Salzman et al., 1990).

**Figure 1:**
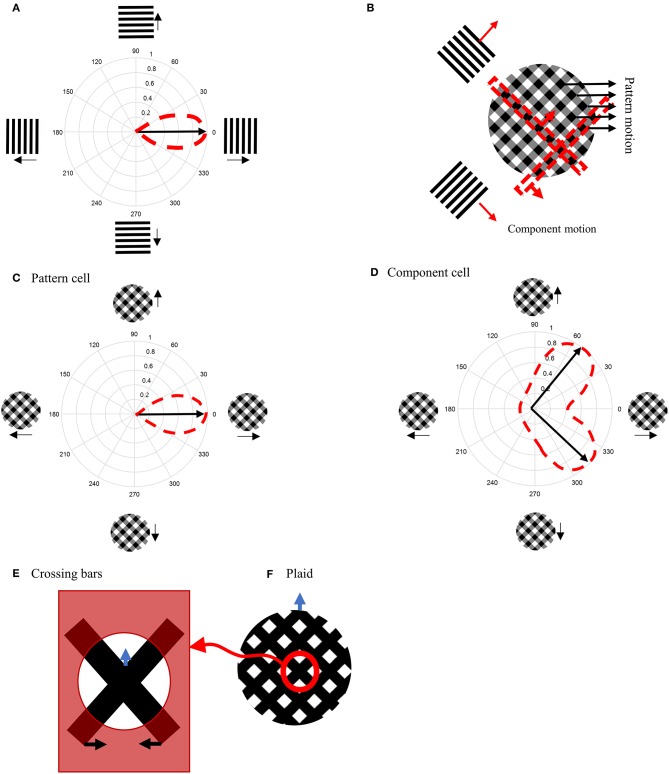
**(A)** Direction-selective neurons respond only to motion in a restricted set of directions. **(B)** A plaid stimulus is formed by the summation of the motion of two gratings moving in different directions. The motion of the plaid is equivalent to the local motions at the extrinsic terminators, which are formed at the intersections of the gratings. In a plaid stimulus, the actual endpoints of the gratings (intrinsic terminators) are hidden. **(C)** The directional tuning curve of a pattern cell. A pattern cell is selective for the direction of the pattern motion of the plaid and has a single lobed directional tuning function centered on the pattern direction. **(D)** The tuning curve of a component cell. A component cell shows two peaks in its directional tuning function, each representing the motion of the individual gratings within a plaid. **(E)** Crossing bars with occluded intrinsic terminators. The local motion of the crossing bars at the intersections represent the pattern motion of a plaid **(F)**.

When a bar or grating is moved through the receptive field (RF) of an MT neuron, it responds only to a restricted range of directions orthogonal to the grating's orientation, making the cell direction selective (Figure 1A). If two gratings with different orientations are presented simultaneously and moved in directions orthogonal to their orientations, a plaid stimulus is created (Figure 1B). Humans perceive plaids as a global motion of the pattern in the direction of the motion of the intersection points of the gratings (indicated by the black arrows in Figure 1B).

When MT neurons are stimulated with plaid patterns, a range of cell-specific responses are observed. About one third of MT neurons, referred to as “pattern” cells, are selective to the direction of the pattern motion and have a single-lobed directional tuning function centered on the pattern direction (Figure 1C). At the opposite end of the spectrum are “component” MT cells, which do not respond optimally to the unified plaid pattern direction but rather show two peaks in their directional tuning functions, each peak coinciding with the direction of the component gratings (Figure 1D). The final third of cells have intermediate properties: they produce broad directional tuning functions that do not have significant double peaks (Albright, 1984; Rodman and Albright, 1989; Movshon et al., 1992; Li et al., 2001).

Several models have been proposed to explain the pattern selectivity of MT neurons wherein the mechanism to produce pattern selectivity employs a hierarchy of processing from component to pattern selective MT cells within MT area itself (Rust et al., 2006). In most models, the extraction of the correct direction of motion for differently oriented bars that move over each other (crossing bars, Figure 1E), has required feedback circuits from neurons in the medial superior temporal (MST) area (Grossberg, 1994, 2014, 2015; Berzhanskaya et al., 2007).

Here, we propose a biologically plausible model of MT that uses as inputs the known properties of three types of cells in the proceeding visual area, primary visual cortex (V1). The novelty of this model is that it is the relative proportions of the inputs from these three types of V1 neurons that determine the component or pattern selectivity of the MT neurons. The model provides a simple mechanism by which component and pattern selective cells can be created that does not require a hierarchical relationship between component and pattern MT cells. The presented model solves several of the problems associated with MT motion detection, such as overcoming the aperture problem (Born and Bradley, 2005; Bradley and Goyal, 2008) and extracting the correct motion directions from crossing bars (Blake and Troscianko, 1990). The model inherently explains several important characteristic properties of pattern MT neurons, including their temporal dynamics (Smith et al., 2005, 2009), the contrast dependency of pattern selectivity (Stoner and Albright, 1992; Kumbhani et al., 2008), and the spatial and temporal limits of pattern motion detection (Majaj et al., 2007; Kumbhani et al., 2015).

## Methods

**Figure 2:**
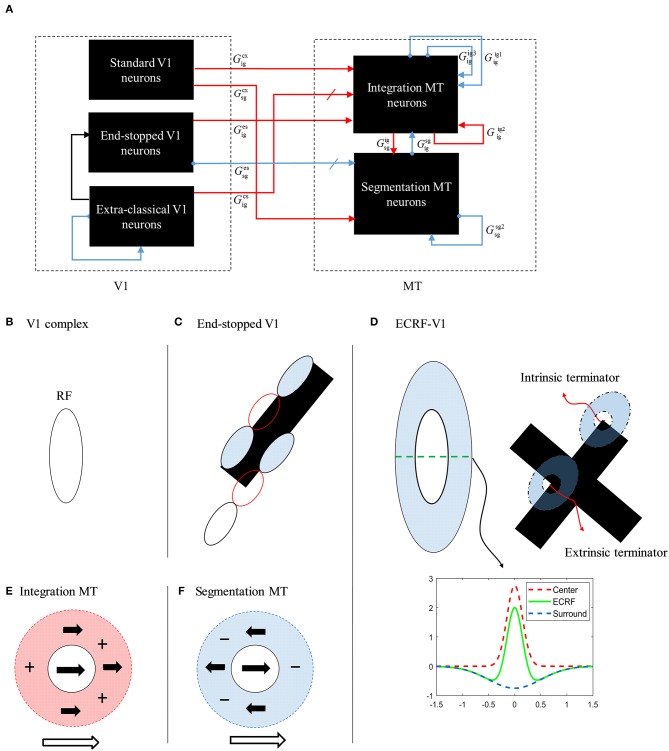
**(A)** A schematic diagram showing the interconnections of the neurons in MT and V1 in the model. Red arrows represent excitatory interconnections between neurons and blue lines indicate inhibitory connections. Black solid lines indicate the effect of V1-ECRF neurons on the threshold level defined by the activity of end-stopped neurons for the inhibitory connections between neurons. **(B)** The receptive field of a V1 complex neuron. **(C)** The receptive field of end-stopped V1 neurons. End-stopped neurons respond strongly to the terminators. The activities of the end-stopped neurons are suppressed in response to long edges because of the inhibitory interconnections from neighboring neurons. **(D)** The receptive fields of V1-ECRF neurons. The activity of the V1-ECRF neurons are suppressed at the extrinsic terminators where their inhibitory surround is more highly activated compared to the intrinsic terminators. The receptive field of these neurons is modeled as differential Gaussians. The subplot shows this function only in one dimension. **(E)** The receptive field of integration MT neurons. The integration MT neurons have facilitatory surrounds. Surround motion in the preferred direction of the center has an excitatory effect, and motion in the opposite direction is inhibitory. **(F)** The receptive field of segmentation MT neurons. The surround region of the segmentation MT neurons has a suppressive effect. The neurons respond well when there is a discontinuity in motion between the center and surround.

We start by providing a brief overview of the model, which is illustrated in Figure 2, followed by a detailed description of the response characteristics of its component parts in later sections. The model processes information in two stages that each incorporate cells with response properties based on those readily found in primate V1 and MT (Albright, 1984; Rodman and Albright, 1989; Movshon et al., 1992; Li et al., 2001). The initial motion and form information are extracted by neurons in V1 by three different types of neurons with distinctive response characteristics: complex V1 neurons, end-stopped V1 neurons, and complex V1 neurons that have suppressive, Extra-Classical RFs (V1-ECRF). The complex V1 and end-stopped V1 neurons are both orientation-selective and direction-selective, the directional preferences being orthogonal to the preferred orientation selectivity (Figure 2D). The central, classical RFs of the V1-ECRF neurons are orientation-selective but not direction-selective. Complex V1 neurons provide initial motion information that signals the direction of motion of the borders of the stimuli. End-stopped neurons respond most strongly to the unambiguous motion information provided by the ends of oriented structures (intrinsic terminators) or the crossing points of multiple contours (extrinsic terminators). The terminators provide cues to resolve the aperture problem in MT (Zarei Eskikand et al., 2016). The amplitudes of V1-ECRF neurons are modulated by the relative amount of texture in their extra-classical RF surrounds. As we will show, the V1-ECRF neurons provide useful information about the form of the stimulus. The resulting visual information is transmitted to the neurons in MT for integration of local motion signals and segregation of overlapping stimuli. The overall model is illustrated in Figure 2A and builds upon the model described by Zarei Eskikand et al. (2018).

The model has two types of MT neurons (Zarei Eskikand et al., 2016). Integration MT neurons are excited by motion in their preferred directions in the center of their RFs. Motion in the preferred direction in the RF surround does not directly induce excitation. However, if preferred direction motion is present in both the center and surround, the response of the cell is higher than for center-stimulation alone. These cells have been identified in MT (Huang et al., 2007). In our model, integration neurons propagate unambiguous motion signals from the terminators in the stimulus to other parts of the MT neural network.

Segmentation MT neurons are excited by motion in one direction in the centers of their RFs and suppressed by motion in the opposite direction in the surrounds, and *vice versa*. The suppressive effect of the surround in the segmentation neurons has an important role in detecting motion discontinuities in the input stimulus (Zarei Eskikand et al., 2016); these cells are common in MT (Huang et al., 2007, 2008). It has been shown that MT neurons can switch between integration and segmentation configurations (Huang et al., 2007; Thiele, 2007) and this dynamic characteristic can be readily incorporated into our model but we do not present this additional computation here.

### V1 Neurons

Initial motion information is extracted by V1 neurons, which have small RFs. The RFs of these neurons are spatiotemporal filters that are modeled using motion energy filters (Adelson and Bergen, 1985; van Santen and Sperling, 1985). The spatial filters of these motion energy filters are Gabor functions and the temporal filters are multi-stage low-pass filters,

(1) $$\begin{array}{r} g_{n}\left(t\right)={\left(t/\tau_{g}\right)}^{n}\exp\left(-t/\tau_{g}\right)\;\left[\frac{1}{n!}-\frac{{\left({t/\tau }_{\text{g}}\right)}^{2}}{\left(n+2\right)!}\right]. \end{array}$$

where τ_*g*_ is the time constant of the filter and *n* takes different values of 6 and 9 simulating the delay between two different temporal filters to compute the motion of the stimulus. These parameters of the temporal filter adjust the velocity selectivity of the neurons. For simplicity, we only consider neurons tuned to the same speed of motion. The outputs of the spatiotemporal filters are combined to form motion energy filters (Zarei Eskikand et al., 2016).

V1 neurons detect only the components of the motion signals that are orthogonal to the edges of the stimulus because of their small RFs, which results in the aperture problem (Born and Bradley, 2005). However, neurons at the terminators (ends) of oriented contours provide unambiguous motion signals because the corners are two-dimensional. To suppress the ambiguous activity of V1 neurons along the long-edges, a model of end-stopped V1 neurons is essential that responds most strongly to the terminators of the stimulus (Hubel and Wiesel, 1965; Pack et al., 2003; Zarei Eskikand et al., 2018). The activity of these neurons is affected by the inhibitory interconnections between neurons, which are effective only if the neighboring neurons have activity above a threshold level (Tsui et al., 2010). The activity of an end-stopped V1 neuron is computed as

(2) $$\begin{array}{l} \frac{d}{dt}v_{x,y,\theta }^{\text{es}}(t)=\left(1-v_{x,y,\theta }^{\text{es}}(t)\right)\left(G_{\text{es}}^{\text{cx}1}v_{x,y,\theta }^{\text{cx}}(t)\right)\\ \;-v_{x,y,\theta }^{\text{es}}\left(\tau_{\text{es}}+G_{\text{es}}^{\text{cx}2}\Gamma_{x,y,\theta }(t)\right), \end{array}$$

where $v_{x,y,\theta }^{\text{es}}$ is the activity of an end-stopped cell selective to direction *θ* (eight different directions: 0°, 45°, 90°, …) located at the coordinate (*x, y*), $v_{x,y,\theta }^{\text{cx}}$ is the activity of the complex neuron in the same location and direction, τ_*es*_ is a decay rate, and $G_{\text{es}}^{\text{cx}1}$ and $G_{\text{es}}^{\text{cx}2}$ are constant gains. The parameter *Γ*_*x, y*, *θ*_ is the inhibition that the neuron receives from complex V1 neurons when the activity of the neighboring standard complex neurons is above the threshold, $\rho_{x,y}^{\text{cx}}$,

(3) $$\Gamma_{x,y,\theta }=\{\begin{array}{l} \sum_{i=-8}^{8}\sum_{j=-8}^{8}\mu_{i,j}\;v_{x+i,y+j,\theta }^{\text{cx}}\;{v_{\text{x}+\text{i,y}+\text{j,}\theta }^{\text{cx}}|}_{i=-3,j=-3}^{i=3,j=3}>\rho_{x,y}^{\text{cx}}\\ 0\text{ otherwise}, \end{array}$$

where *μ*_*i, j*_is the inhibitory connectivity matrix that extends across a patch of 8x8 neighboring neurons and has a discretized Gaussian shape (Zarei Eskikand et al., 2016).

The activities of end-stopped V1 neurons at the intrinsic terminators carry unambiguous motion information, but their responses to extrinsic terminators, formed when objects overlap, conflict with the global movement of the stimulus. Therefore, we have incorporated input from V1-ECRF neurons that are orientation-selective but also have contrast-sensitive suppressive surrounds.

The V1-ECRF neurons receive a suppressive effect from their surrounds, which is activated by the luminance of the stimulus. The activities of the neurons are modeled as the difference of Gaussians (also known as the Mexican Hat function),

(4) $$\begin{array}{r} R_{x,y,o}=A_{C}\exp\left(-\left(\frac{{x_{o}}^{2}}{\sigma_{xc}^{2}}+\frac{{y_{o}}^{2}}{\sigma_{yc}^{2}}\right)\right)-A_{S}\exp\left(-\left(\frac{{x_{o}}^{2}}{\sigma_{xs}^{2}}+\frac{{y_{o}}^{2}}{\sigma_{ys}^{2}}\right)\right) \end{array}$$

where (*x*_*o*_,*y*_*o*_) are oriented coordinates with orientation *o* at spatial location (*x*,*y*), $\sigma_{xc}^{2}$ and $\sigma_{yc}^{2}$ are the standard deviations of the centers of the RF. The parameters $\sigma_{xs}^{2}$ and $\sigma_{ys}^{2}$ are standard deviations of the surround and *A*_*C*_ and *A*_*S*_ are constants. The RF of the V1-ECRF neurons is applied to the reversed intensity level of the input stimulus, *I*_*x, y*_, to compute their activities,

(5) $$\begin{array}{r} v_{x,y,o}^{\text{cs}}=h\left(R_{x,y,o}*I_{x,y}\;\right) \end{array}$$

where *h*(.) is a saturating linear function

(6) $$h_{\left(x\right)}=\{\begin{array}{ll} 1, & \text{if }x\geq 1\\ x, & \text{if }0\leq \text{x }<\;1\\ 0, & \text{if x }<\;0 \end{array}$$

that is applied to the result of the two-dimensional convolution, symbolized by ^*^, that keeps the activity between 0 and 1. The superscript “*cs*” stands for V1-ECRF neurons. This resulting initial form information is transmitted to MT neurons by the interaction of V1-ECRF neurons and complex V1 neurons. The activity level of complex V1 neurons is gated by the V1-ECRF neurons,

(7) $$\kappa_{x,y,\theta }=\{\begin{array}{ll} v_{x,y,\theta }^{\text{cx}} & \text{if }v_{x,y,o}^{\text{cs}}>0\\ 0 & \text{otherwise}, \end{array}$$

where $\kappa_{x,y,\theta }$ is the effect of the form information provided by the V1-ECRF neurons on neurons responding to the motion information at location (*x, y*) selective to direction *θ*, $v_{x,y,\theta }^{\text{cx}}$ is the activity of complex V1 neurons, and $v_{x,y,o}^{\text{cs}}$ is the activity of the V1-ECRF neurons at the orientation, *o*, that is orthogonal to the direction, *θ*. It is necessary for complex V1 neurons to interact with V1-ECRF neurons that have orientation selectivity orthogonal to their direction preferences because motion-detecting neurons respond to the changes of the intensity level orthogonal to the edge of the moving stimulus. The intended effect is that the inputs to MT from complex V1 neurons have a high level of motion information only where the V1-ECRF neurons are active. Therefore, $\kappa_{x,y,\theta }$ is inactive at the extrinsic terminators where the activity of V1-ECRF neurons is suppressed.

### MT Neurons

The initial motion signals computed by V1 neurons are transmitted to the MT neurons. The integration MT neurons propagate the motion information from the regions with stronger activity of V1 neurons to other regions via recurrent excitatory interconnections. The presence of the end-stopped V1 neurons results in higher levels of neural activity at the terminators compared to ambiguous motion information at the edges of the stimulus, which would otherwise create the aperture problem. Therefore, unambiguous motion signals generated at the terminators win over the other motion directions and gradually dominate the motion direction to which the MT neurons respond.

Integration neurons receive excitatory input from the luminance sensitive V1-ECRF neurons, whose activities are suppressed at the extrinsic terminators. Thus, the connection strength from the V1-ECRF neurons to MT neurons determines the impact of the extrinsic terminators on the perceived direction of motion by MT neurons compared to intrinsic terminators. The activity of the integration neurons is computed by

(8) $$\begin{array}{l} \frac{dv_{x,y,\theta }^{\text{ig}}(t)}{dt}=h(G_{\text{ig}}^{\text{cx}}v_{x,y,\theta }^{\text{cx}}(t)+G_{\text{ig}}^{\text{es}}v_{x,y,\theta }^{\text{es}}(t)+G_{\text{ig}}^{\text{ig2}}\lambda_{x,y,\theta }(t)+G_{\text{ig}}^{\text{cs}}\kappa_{x,y,\theta }(t)-\\ \;G_{\text{ig}}^{\text{ig1}}\gamma_{x,y,\theta }(t-T^{\text{ig}})-G_{\text{ig}}^{\text{ig3}}\zeta_{x,y,\theta }(t-T^{\text{ig}})-G_{\text{ig}}^{\text{sg}}v_{x,y,\theta }^{\text{sg}}(t)-\tau_{\text{ig}}v_{x,y,\theta }^{\text{ig}}(t)), \end{array}$$

where λ_*x, y*, *θ*_ is the excitation from neighboring integration neurons selective to the same direction *θ*, γ_*x, y*, *θ*_ is inter-directional inhibition, and ζ_*x, y*, *θ*_ is long-range inhibition. These neurons receive excitatory input from complex V1 and end-stopped V1 neurons, $v_{x,y,\theta }^{\text{cx}}$ and $v_{x,y,\theta }^{\text{es}}$, respectively. They also receive inhibitory input from segmentation neurons, $v_{x,y,\theta }^{\text{sg}}$. Finally, *h*() is the piece-wise linear saturation function that constrains the level of activity to between 0 and 1, defined in Equation (6). $G_{\text{ig}}^{\text{cs}}$ is the variable parameter that determines the pattern motion selectivity of the MT neurons. The remaining excitatory and inhibitory connection strengths are given by constant gain factors, $G_{\text{ig}}^{\text{cx}}$, $G_{\text{ig}}^{\text{es}}$, $G_{\text{ig}}^{\text{ig2}}$, $G_{\text{ig}}^{\text{ig1}}$, $G_{\text{ig}}^{\text{ig3}}$, $G_{\text{ig}}^{\text{sg}}$, whose values are given in Table 1. *T*^ig^ indicates the time delay of inhibition between MT integration neurons and τ_ig_ is the decay rate of the activity of integration MT neurons. The receptive field areas of MT neurons are seven times larger than the receptive fields of V1 neurons, and the area of the surrounds of MT neurons are ten times larger than the receptive fields of V1 neurons (Zarei Eskikand et al., 2016).

**Table 1:** The constant parameters used in the model, their values, and their units.

**Description**	**Parameter**	**Value**	**Unit**
Connection strength of input to the end-stopped neurons	${\text{G}}_{\text{es}}^{\text{cx}1}$	2	–
Connection strength of inhibitory connections on end-stopped neurons	${\text{G}}_{\text{es}}^{\text{cx}2}$	3	–
Connection strength of complex V1 neurons to integration neurons	${\text{G}}_{\text{ig}}^{\text{cx}}$	0.5	–
Connection strength of end-stopped V1 neurons to integration neurons	${\text{G}}_{\text{ig}}^{\text{es}}$	0.7	–
Connection strength of complex V1 neurons to segmentation neurons	${\text{G}}_{\text{sg}}^{\text{cx}}$	1	–
Connection strength of excitatory connections to integration neurons	${\text{G}}_{\text{ig}}^{\text{ig}2}$	0.2	–
Connection strength of inter-directional inhibitory connections	${\text{G}}_{\text{ig}}^{\text{ig}1}$	1	–
Connection strength of long-range inhibitory connections	${\text{G}}_{\text{ig}}^{\text{ig}3}$	1	–
Connection strength of inhibition from segmentation neurons	${\text{G}}_{\text{ig}}^{\text{sg}}$	0.1	–
Connection strength of excitation from integration on segmentation neurons	${\text{G}}_{\text{sg}}^{\text{ig}1}$	0.5	–
Connection strength of surround facilitation on segmentation neurons	${\text{G}}_{\text{sg}}^{\text{sg}1}$	0.1	–
Connection strength of surround suppression on segmentation neurons	${\text{G}}_{\text{sg}}^{\text{sg}2}$	0.2	–
Number of neurons at each location-selective to different directions	*N*	8	–
Constant value for the threshold on the activity of complex V1 neurons	c^cx^	0.12	
Constant value for the threshold on the activity of segmentation neurons	c^sg^	0.02	–
Constant value for the threshold on the activity of integration neurons	c^ig^	0.05	–
Decay rate of the activity of integration neurons	τ_ig_	0.2	–
Decay rate of the activity of segmentation neurons	τ_sg_	0.2	–
Decay rate of the activity end-stopped neurons	τ_es_	0.01	–
Simulation time step	Δ*t*	0.01	ms
Time constant of the temporal filter	τ_g_	0.01	ms
Time delay of inhibition between MT integration neurons	T^ig^	0.1	ms
Spatial frequency	*f*	1.1	cyc/deg
Standard deviation of horizontal spatial Gaussian filter	σ_x_	0.5	–
Standard deviation of vertical spatial Gaussian filter	σ_y_	0.5	–
Standard deviation of center portion of horizontal spatial Gaussian filter	σ_xc_	0.35	–
Standard deviation of center portion of vertical spatial Gaussian filter	σ_yc_	0.4	–
Standard deviation of surround portion of horizontal spatial Gaussian filter	σ_xs_	0.4	–
Standard deviation of surround portion of vertical spatial Gaussian filter	σ_ys_	0.5	–
Strength of center portion of spatial Gaussian filter	*A_*c*_*	1	–
Strength of surround portion of spatial Gaussian filter	*A_*s*_*	0.72	–

Segmentation MT neurons constrain the propagation of motion signals to the regions where there is movement by detecting discontinuities in the stimulus and inhibiting the activity of the integration neurons in these regions. These inhibitory connections also prevent the integration of motion signals generated by multiple moving stimuli. Complex V1 neurons provide the initial motion signals and border information of the moving stimulus to the segmentation neurons. Segmentation neurons receive inhibitory connections from end-stopped V1 neurons to prevent interference with the propagation of the unambiguous motion signals at the terminators. The strength of these inhibitory connections has a key role in determining the level of the pattern motion selectivity of the MT neurons. Strong inhibition from end-stopped V1 neurons suppresses the activity of the segmentation neurons at the extrinsic terminators. Thus, extrinsic terminators have a stronger impact on the perceived motion of the input stimulus, which eventually results in pattern motion selectivity of the MT neurons. However, decreasing the inhibitory effect of the end-stopped neurons on segmentation neurons causes higher activity levels of the segmentation neurons at the terminators, which results in suppression of the integration neurons through inhibitory input due to the extrinsic terminators. Therefore, decreasing the impact of the motion signals at the extrinsic terminators enhances the component motion selectivity of the MT neurons. The activity of the segmentation neurons is given by

(9) $$\begin{array}{l} \frac{dv_{x,y,\theta }^{\text{sg}}}{dt}=h(G_{\text{sg}}^{\text{cx}}v_{x,y,\theta }^{\text{cx}}-G_{\text{sg}}^{\text{es}}v_{x,y,\theta }^{\text{es}}+G_{\text{sg}}^{\text{ig}}\eta_{x,y}+G_{sg}^{sg1}\chi_{x,y,\theta }^{\text{e}}\\ \;-G_{\text{sg}}^{\text{sg}2}\chi_{x,y,\theta }^{\text{i}}-\tau_{\text{sg}}v_{x,y,\theta }^{\text{sg}}), \end{array}$$

where *η*_*x, y*_ is the excitatory input received from integration neurons and χ_*x, y*, *θ*_ represents the excitatory and inhibitory interconnections between segmentation neurons as the result of center-surround interactions. $G_{\text{sg}}^{\text{es}}$ is a variable parameter that is altered within a limited range to investigate the level of pattern motion selectivity of the MT neurons. All remaining parameters are constant and their values are given in Table 1. Further details about the roles of the parameters can be found in Zarei Eskikand et al. (2016).

To investigate the level of pattern motion selectivity of the modeled MT neurons, we examined the model response when two overlapped moving bars have occluded intrinsic terminators. To simulate pattern motion, we removed the occluders from the RFs of all neurons by limiting the image frame to the intersections of the bars.

The responses of MT neurons to pattern or component motions are determined by the values of two parameters of the model: the connection strength of the excitatory input from V1-ECRF neurons, $G_{\text{ig}}^{\text{cs}}$, and the inhibitory effect of end-stopped V1 neurons, $G_{\text{sg}}^{\text{es}}$. The values of these parameters determine whether the MT neuron is selective to pattern motion or to component motion. To measure the level of pattern motion selectivity compared to component motion selectivity, we used pattern index, *P*^I^, which represents the level of the pattern direction selectivity of the MT neurons depending on the relative strengths of the connections from end-stopped V1 neurons and V1-ECRF neurons, defined as

(10) $$\begin{array}{r} {P^{\text{I}}|}_{G_{\text{ig}}^{\text{cs}},G_{\text{sg}}^{\text{es}}}={S^{\text{P}}|}_{G_{\text{ig}}^{\text{cs}},G_{\text{sg}}^{\text{es}}}-{S^{\text{C}}|}_{G_{\text{ig}}^{\text{cs}},G_{\text{sg}}^{\text{es}}}. \end{array}$$

${S^{\text{P}}|}_{G_{\text{ig}}^{\text{cs}},G_{\text{sg}}^{\text{es}}}$is the level of the pattern motion selectivity of MT neurons, and its value depends on two parameters of $G_{\text{ig}}^{\text{cs}}$and $G_{\text{sg}}^{\text{es}}$,

(11) $$\begin{array}{r} {S^{\text{P}}|}_{G_{\text{ig}}^{\text{cs}},G_{\text{sg}}^{\text{es}}}=\left(1-N\left(\underset{x}{\sum }\underset{y}{\sum }{v_{x,y,\theta }^{\text{ig}}|}_{G_{\text{ig}}^{\text{cs}},G_{\text{sg}}^{\text{es}}}-v_{x,y,\theta }^{dp}\right)\right), \end{array}$$

where $v_{x,y,\theta }^{dp}$ is the expected activity of the neurons responding to the pattern motion of the stimulus and $v_{x,y,\theta }^{\text{ig}}$ is the activity of the MT neuron at location (*x, y*) when the strengths of the inhibitory connections from end-stopped V1 neurons and V1-ECRF neurons are $G_{\text{sg}}^{\text{es}}$ and$G_{\text{ig}}^{\text{cs}}$, respectively. *N* is a normalization function that scales all values between 0 and 1 described as *N*(*v*) = (*v* − min(*v*))/(max(*v*) − min(*v*)). ${S^{\text{C}}|}_{G_{\text{ig}}^{\text{cs}},G_{\text{sg}}^{\text{es}}}$ is the level of the component direction selectivity of the MT neurons,

(12) $$\begin{array}{l} {S^{\text{C}}|}_{G_{\text{ig}}^{\text{cs}},G_{\text{sg}}^{\text{es}}}=1-N(\sum_{x}\sum_{y}\left({v_{x,y,\theta_{1}}^{\text{ig}}|}_{G_{\text{ig}}^{\text{cs}},G_{\text{sg}}^{\text{es}}}-v_{x,y,\theta_{1}}^{dc}\right)\\ \;+\sum_{x}\sum_{y}{(v_{x,y,\theta_{2}}^{\text{ig}}|}_{G_{\text{ig}}^{\text{cs}},G_{\text{sg}}^{\text{es}}}-v_{x,y,\theta_{2}}^{dc})), \end{array}$$

where $v_{x,y,\theta_{1}}^{\text{ig}}$and $v_{x,y,\theta_{2}}^{\text{ig}}$are the activities of the integration MT neurons selective to the directions of the first and second components of the stimulus, respectively, and $v_{x,y,\theta_{1}}^{dc}$ and $v_{x,y,\theta_{2}}^{dc}$ are the desired activities of the integration MT neurons responding to the first and second component motions, respectively.

To investigate the effects of changes in contrast, the model is tested with two overlapping bars with different contrast values, again with occluded intrinsic terminators. The effects of changing the contrast level of the stimulus on the pattern motion selectivity of MT neurons are investigated.

## Results

To investigate the response of the model presented here, we used crossing bars as stimuli (Figure 1E). The bars are slanted left and right at 45° from the vertical; the bars move horizontally either left or right, respectively. When the intrinsic terminators of the crossing bars (i.e., the ends of the bars) are occluded, the extrinsic terminators formed by the crossing of the bars becomes the dominant driver of perception (Figure 1F). Human observers perceive this pattern as moving upwards.

Crossing bars resemble the motion of a plaid in the absence of their intrinsic terminators. The plaid in Figure 1B is constructed by summing two differently oriented gratings that have relatively low contrasts. At the crossing points, the contrast is higher due to the summing process. The plaid in Figure 1F was created in the same way but both bars have zero luminance, so the crossing points are not darker than the bars (a saturated plaid). Therefore, two crossing bars with different orientations have the same characteristics as the elements of a saturated plaid.

### Responses of V1 Neurons to Crossing Bar Stimuli

**Figure 3:**
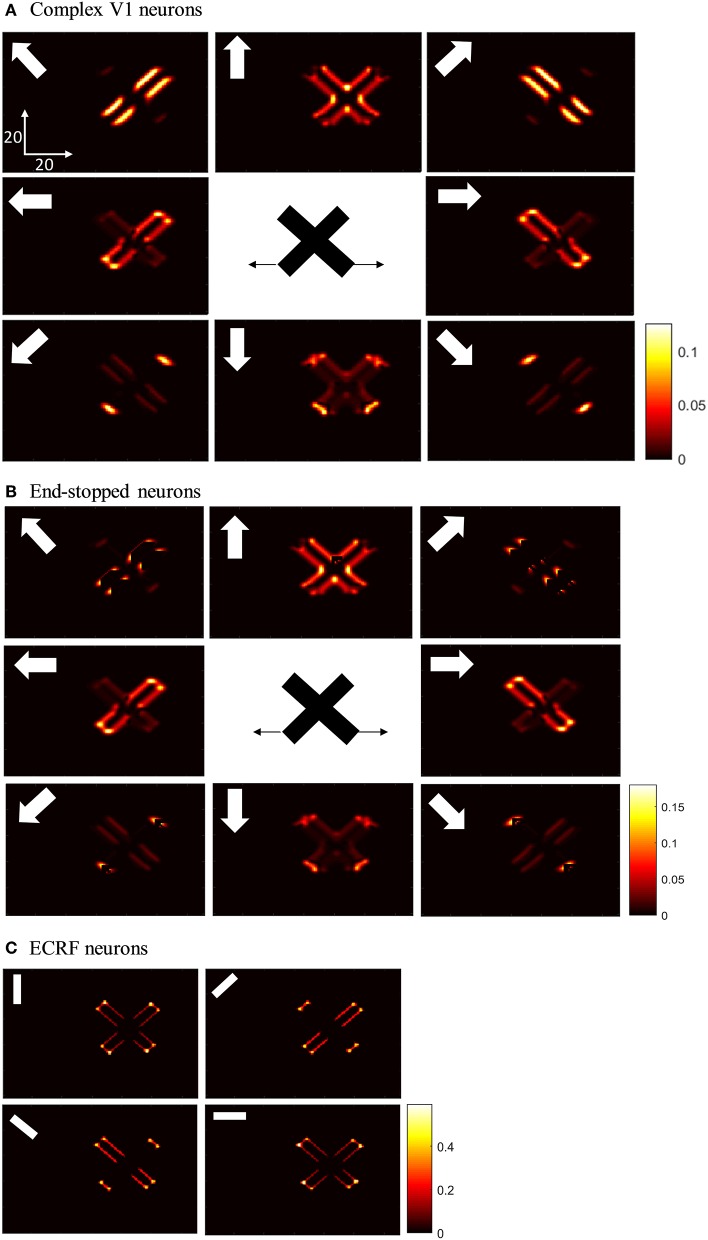
**(A)** The activities of complex V1 neurons selective to eight different directions in response to overlapped moving bars with the same level of contrast. Each box shows the activity of the neurons selective to the direction shown by the white arrow. The axes represent the spatial location. The color bar shows the strength of the activity, brighter for higher values, and the white arrows indicate the preferred direction of the neurons in each graph. The stimulus is two crossing bars with the same level of contrast, as illustrated in the middle of the figure. The bar with 135° orientation is moving to the right and the bar with 45° orientation is moving to the left (black horizontal arrows). The neurons have a high level of activity at the terminators and also along the edges of the bars. **(B)** The activity of V1 end-stopped neurons in response to crossing bars with the same level of contrast. The neurons represent a high level of activity at both the intrinsic and extrinsic terminators. **(C)** The activities of V1-ECRF neurons in response to crossing bars with the same level of contrast. The preferred orientations of the neurons in each graph are shown by the white bars. The neurons have the highest level of activity at the intrinsic terminators and their activities are strongly suppressed at the extrinsic terminators.

The initial motion signals are extracted by phase insensitive, direction-selective, complex V1 neurons. The phase-insensitivity of the neurons and their direction-selectivity are generated by a non-linear combination of spatial and temporal filters (Adelson and Bergen, 1985; van Santen and Sperling, 1985). The small RFs of these neurons result in ambiguous directional information. The activities of the complex V1 neuron network when responding to two moving, crossing bars with the same contrast are shown in Figure 3A. In this and subsequent figures, each of the eight boxes show the responses of neurons in the V1 network. Their preferred direction is represented by the white arrows in the boxes. The neurons selective to the directions perpendicular to the orientations of the bars have high levels of activity when the edges of the bars cross their RFs (top left and top right boxes). These neurons respond in a manner consistent with those affected by the aperture problem. The aperture problem occurs when the RFs of the cells are too small to see anything other than the movement of the edges of the bars through their RFs. As a result, even though the bars are moving horizontally, because of their slanted orientations, within the RFs the edges of the bars move orthogonal to their orientations (Born and Bradley, 2005; Bradley and Goyal, 2008). Neurons that are selective for leftward and rightward motion (middle left and middle right boxes) are most strongly activated when the intrinsic terminators move over their RFs; i.e., the strength of the activity at the corners of the bars are bright yellow (middle left and middle right boxes). These cells give the most accurate estimations of the directions of movement. However, it should be noted that rightward and leftward sensitive V1 complex cells also respond quite strongly along the edges of the bars (diagonal orange patches in the middle left and middle right boxes). V1 complex cells selective to upward motion (middle upper box) respond most strongly when the extrinsic terminators (i.e., the crossing points of the bars) move over their RFs. Based on the outputs of the complex V1 neurons, it is impossible to extract an unequivocal, correct direction of motion from the collective cell population as strong signals are available in different parts of the visual field from cells tuned to different directions.

The end-stopped V1 neurons have the same form as the V1 complex neurons except that their immediate co-linear neighbors provide inhibition so that maximum excitation occurs only when the terminators of the bars match a particular RF (Figure 2C). Therefore, the end-stopped neurons have edge-length-dependent response amplitudes (Hubel and Wiesel, 1965). End-stopped neurons are essential for subsequent MT neurons to differentiate the unambiguous motion information of the terminators from the ambiguous motion information that results from the aperture problem (Zarei Eskikand et al., 2016). Figure 3B shows the activities of end-stopped neurons in response to the same stimulus as used in Figure 3A. The population response is qualitatively similar to that of the standard V1 cells except in one very important respect: the activities of the end-stopped V1 neurons selective to upward-left and upward-right motion (top-left and top-right boxes) generate strong suppression when the edges of the stimulus cross their RFs. Therefore, these cells are far less influenced by the aperture problem.

To discriminate the motion signals of the intrinsic from the extrinsic terminators, in our model, we incorporate orientation-selective V1-ECRF neurons that have extensive suppressive surrounds. In these neurons, the central excitatory region has a similar RF structure to that of the V1-complex cells outlined above. However, the central region in this case is not direction-selective, so the cells signal form rather than motion. A wider inhibitory RF is also incorporated into the RFs of these cells, which is sensitive to the luminance of the stimulus. The surrounds suppress the activities of the neurons when activated by the dark luminance of the stimulus in the case of a white background. The activities of the V1-ECRF neurons are strongly suppressed at the extrinsic terminators where the inhibitory surrounds of the neurons are more highly stimulated than at the intrinsic terminators (Figure 3C). The reason for this is that there is a larger area stimulated by the black bars at the extrinsic terminator locations than at the intrinsic terminator locations (Figure 2D). As the signals from the extrinsic terminators are suppressed, the influence of those signals at the population level is reduced. Therefore, the signals from the intrinsic terminators are able to dominate, thus emphasizing cells that are signaling the correct leftward and rightward directions of the bars. Thus, excitatory connections from neurons at the intrinsic terminators assist the network of MT neurons to highlight the most appropriate motion signals to be used for perception.

### Responses of V1 Neurons With Occluded Intrinsic Terminators

**Figure 4:**
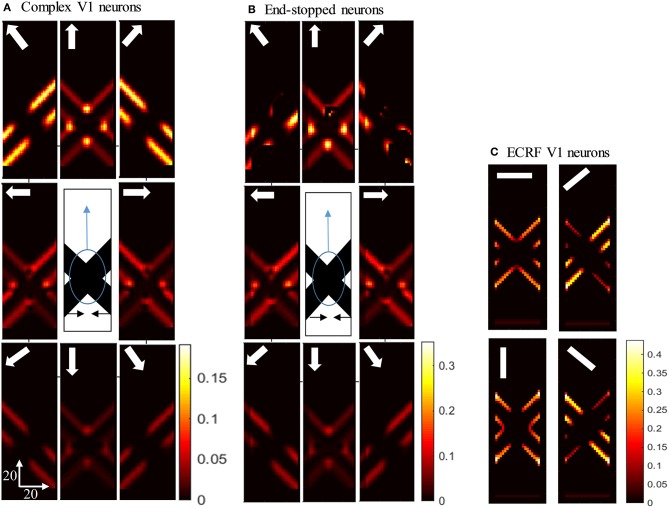
**(A)** The activities of complex V1 neurons responding to the pattern motion. The stimulus (shown in the middle) is two crossing bars with hidden intrinsic terminators. The bars are actually moving to the left and right (horizontal black arrows). Each box shows the activity of the neurons selective to the direction shown by the white arrow. The axes represent the spatial location. The color bar shows the strength of activity, brighter for higher values. The neurons have a high level of activity at the extrinsic terminators and along the edges. **(B)** The activity level of end-stopped neurons responding to the pattern motion. The activity of the neurons along the edges are suppressed. **(C)** The activities of V1-ECRF neurons in response to the pattern motion. The preferred orientation of the neurons at each box is shown by the white bars. The activity of the neurons at the extrinsic terminators are strongly suppressed.

Having shown the preferred stimuli for the three classes of V1 neurons, we now show how those cells respond when we remove the intrinsic terminators, as is the case in plaid patterns (see Figure 1E). We use the same overlapping, moving bars as in the previous section but with the intrinsic terminators occluded (Figure 4). The responses of complex V1 neurons to the stimulus are shown in Figure 4A. Among the neurons selective to the up-right direction (top-right box), neurons with RFs at the edges of the rightward moving bar have high levels of activity. Similarly, among the neurons selective to the up-left direction (top-left box), neurons with RFs at the edges of the leftward moving bar have high levels of activity. The neurons at the extrinsic terminators formed at the crossing junctions of the overlapping bars also have high levels of activity (top-central box); these neurons respond to upward motion of the crossing junction. The activities of end-stopped V1 neurons are shown in Figure 4B. The inhibitory interconnections between end-stopped V1 neurons result in suppression of the activity along the edges. Since the intrinsic terminators are hidden, the end-stopped neurons only have high levels of activity at the extrinsic terminators, representing upward motion of the crossing junction (top-central box). Figure 4C shows the activities of the V1-ECRF neurons. The inhibitory surrounds of these neurons are strongly stimulated by the intensity level at the extrinsic terminators. Therefore, the activities of the neurons at the extrinsic terminators are suppressed because of their surround effect. These neurons have high levels of activity only along the edges of the moving bars (Figure 4C).

### Responses of MT Neurons

The responses of the MT neurons are highly dependent on the values of two parameters: the excitatory input that they receive from the interaction of complex V1 and V1-ECRF neurons, $G_{\text{ig}}^{\text{cs}}$, and the inhibitory effect of the end-stopped neurons on the activity of the segmentation MT neurons, $G_{\text{sg}}^{\text{es}}$ (Figure 2A). Increasing the connection weight of the excitatory input from V1-ECRF neurons to MT neurons suppresses the influence of the extrinsic terminators. Extrinsic terminators have a significant role on the pattern motion selectivity of the MT neurons. Therefore, suppressing their effect causes the neurons to become selective to the component motion signal associated with the overlapping bars (in the absence of the intrinsic terminators) when they receive strong input from V1-ECRF neurons. Decreasing the inhibitory effects of the end-stopped V1 neurons on the segmentation MT neurons results in an increase of the suppression effect of the segmentation MT neurons on the integration MT neurons that have their RFs over the extrinsic terminators. Therefore, the effect of the extrinsic terminators in determining the direction of the motion decreases significantly, which results in strengthening the component motion selectivity of the MT neurons.

**Figure 5:**
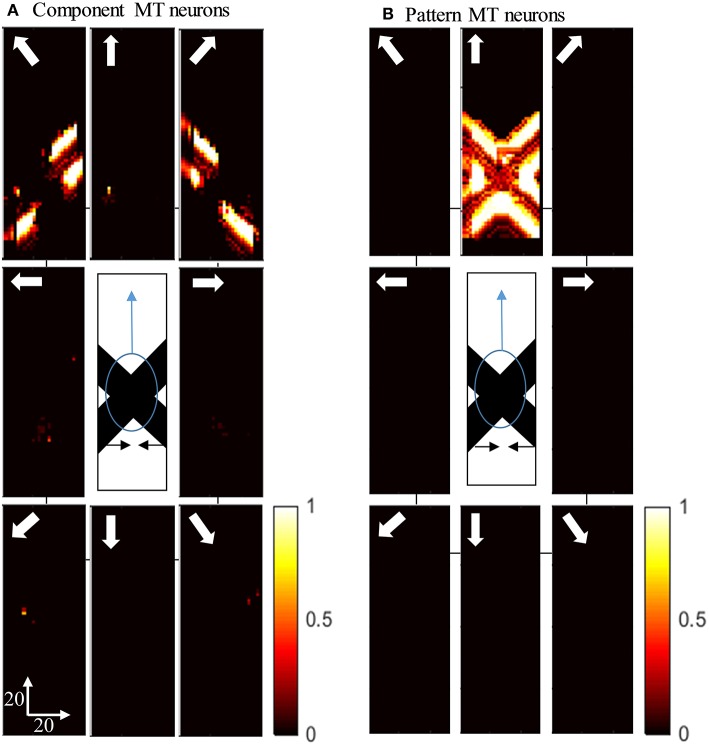
**(A)** The activity of the neurons selective to the component motion in response to the pattern motion. The stimulus is shown in the middle, which is two crossing bars with hidden intrinsic terminators. The bars are actually moving to the left and right (horizontal black arrows). Each box shows the activity of the neurons selective to the direction shown by the white arrow. The color bar shows the strength of activity, brighter for higher values, and the axes represent the spatial location. The neurons selective to the up-right and up-left directions have the highest level of activities representing the component motion of the stimulus. **(B)** The activity of the pattern motion selective MT neurons responding to the pattern motion. The neurons selective to the up direction have the highest level of activities representing the pattern motion of the stimulus.

Figure 5A shows the activity of MT neurons when they are selective to the component motion because of strong input from V1-ECRF neurons and when the inhibitory effect of the end-stopped V1 neurons on segmentation MT neurons is small. The MT neurons represent the component directions, which are the directions of movement of the individual bars when affected by the aperture problem. Neurons respond strongly to the bar with 135^o^ orientation moving in the up-right direction (top-right box), although it is actually moving to the right. Neurons also respond strongly to the bar with 45^o^ orientation moving in the up-left direction (top-left box), although it is actually moving to the left. The pattern motion of the stimulus generated by the extrinsic terminators is suppressed by the excitatory input from the V1-ECRF neurons.

The activities of MT neurons selective for pattern motion are shown in Figure 5B. In this case, the strength of the excitatory connections from V1-ECRF neurons is low, while the inhibitory effect of end-stopped V1 neurons on segmentation MT neurons is increased. The MT neurons propagate the upward motion of the extrinsic terminators along the whole of the stimulus over time, which results in selectivity for pattern motion.

### Pattern Index

**Figure 6:**
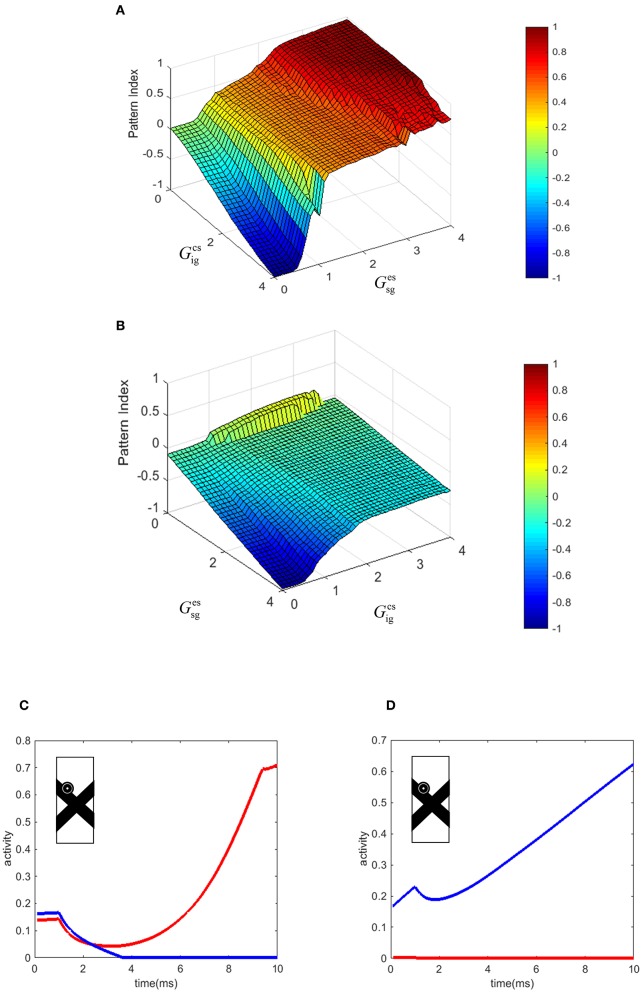
**(A)** The effect of the changes in the connection strength of the V1-ECRF neurons and end-stopped neurons to MT. Positive values of the pattern index represent pattern selectivity of the MT neurons and negative values indicate component selectivity of the MT neurons. The color bar shows the value of the pattern index, brighter for higher values. **(B)** The effect of the changes in the connection strength of the end-stopped and V1-ECRF neurons to MT in response to the pattern motion when the contrasts of the bars are different. **(C)** The temporal dynamic activity of pattern MT neurons. The eyeball circle shows the location of the selected MT neuron in the visual field. The activity of the pattern neuron selective to the upward direction (red line) is lower than the activity of the MT neuron selective to the up-right direction (blue line) at the beginning. After a delay, the neuron selective to the direction of the pattern motion dominates over the neuron selective to the direction of the component motion. **(D)** The temporal dynamic activity of the component MT neuron. The activity of the component neuron selective to the up-right direction (blue line) is higher than the activity of the neuron selective to the upward direction (red line). There is no delay in detecting the component motion of the stimulus in contrast to the pattern motion. The eyeball circle shows the location of the selected MT neuron.

In our model, the level of pattern and component motion selectivity of the MT neurons depends on the relative strengths of the connections from end-stopped V1 neurons and V1-ECRF neurons. To investigate the effects of these connections on the value of the pattern index (Equation 10, see Methods), the corresponding parameters of the model were changed within a limited range. Figures 6A,B show the effects of changing the two parameters of the model on the pattern and component motion selectivity of MT neurons. The negative values (blue) of the pattern index indicate component motion selectivity and positive values (red) indicate pattern selectivity of MT neurons.

Increasing the effect of V1-ECRF neurons, $G_{\text{ig}}^{\text{cs}}$, results in suppression of the signals derived from the extrinsic terminators in determining the motion direction of the stimulus. Therefore, the neurons show preference for the component motion of the input stimulus. An increase in the connection strength from end-stopped V1 neurons to segmentation MT neurons, $G_{\text{sg}}^{\text{es}}$, results in more positive values for pattern selectivity. As the purpose of the inhibitory connections from end-stopped V1 neurons to segmentation MT neurons is to prevent the interference of segmentation neurons in the propagation of the activity from the terminators, increasing the strength of this connection enhances the effect of the extrinsic terminators on determining the motion selectivity of MT neurons. Therefore, increasing this parameter of the model increases the pattern motion selectivity of MT neurons.

### Contrast Dependency of the Pattern Motion Selectivity of MT Neurons

To investigate the effects of changes in the image contrast on the dependency of the pattern index on the strength of these connections, the model was tested when the contrasts of the bars were different. The strengths of the connections from end-stopped V1 and V1-ECRF neurons to MT was changed within a limited range to investigate their effects on the pattern and component motion selectivity of the neurons. Figure 6B shows the pattern index when the model is tested with the stimuli in which the low contrast bar appears to move in front of a high contrast bar.

Similar to the results of the model in response to stimuli with similar contrast levels, increasing the connection strength of the input from V1-ECRF neurons decreases the effect of the extrinsic terminators and increases the component selectivity of the MT neurons. The level of the pattern selectivity of MT neurons drops substantially when the contrast of the bars is different. Therefore, changing the value of the parameters does not have a substantial effect on the pattern selectivity of the neurons. This is consistent with experimental findings that have shown a drop in the pattern selectivity of MT neurons when the contrast of the gratings is different (Stoner and Albright, 1992; Kumbhani et al., 2008).

### Temporal Dynamics of the Response of MT Neurons

There is a delay in the final response of MT neurons to the pattern motion because of the time taken for the population of MT neurons to propagate the activity of the neurons at the extrinsic terminators to cover the whole of the stimulus (Figure 6C). Conversely, component MT neurons only produce high activity along the edges of the stimulus. As this is simultaneous for all cells along the edges, there is no delay in the computation, so the responses do not evolve over time (Figure 6D). This difference in timing between pattern and component neurons has been observed experimentally in primate MT (Smith et al., 2005).

Figure 6C shows the temporal dynamics of detecting pattern motion compared to component motion of an MT neuron in response to the crossing bars with the same level of contrast. The RF of the neuron is located on the edge of the bar. A pattern neuron (Figure 6C) responds to the direction of the component motion at response onset. After a delay, the MT neuron responds to the pattern motion of the stimulus when the motion information has had enough time to propagate from the extrinsic terminators along the whole of the stimulus. However, the component neuron (Figure 6D) does not have a similar temporal dynamic and its response to the component motion dominates over the pattern motion from motion onset. To decrease the computational load, we set a very small value for the time step. This has scaled the final amount of the temporal delay.

## Discussion

Our computational model describes a biologically plausible mechanism that could generate pattern and component motion selectivity in primate MT neurons. The results of the model show that the extrinsic terminators formed at the intersections of overlapping bars have a particularly important role in pattern motion detection in MT neurons. The model uses several known V1 cell types and two types of MT neurons in an integrated network to accentuate the terminators in the moving image. It is this characteristic that allows the model to solve the aperture problem rather than just the integration of component signals from direction-selective V1 cells. The motion information generated at the extrinsic terminators within a plaid represents the direction of the pattern motion. The interconnections between MT neurons propagate the activities of the neurons that respond to the extrinsic terminators to other neurons in the network that are viewing other regions of the stimulus. Pattern selective MT neurons respond to the component motions at the onset of the stimulus and their responses evolve over time to represent the pattern motion of the stimulus as the motion information gradually propagates from the extrinsic terminators.

Neurophysiological findings have revealed the spatial and temporal limits of pattern motion perception in MT neurons (Majaj et al., 2007; Kumbhani et al., 2015). They have shown that there is a requirement for the appearance of the gratings simultaneously in a limited temporal and spatial range for pattern motion detection to occur in MT neurons. These data are consistent with the idea that processing of extrinsic terminators might be a key factor in the generation of pattern motion selectivity in MT neurons. The time between the appearances of the gratings should not exceed the decay rate of the MT responses to the extrinsic terminators in the input stimulus, which are the key elements for the computation of pattern motion.

Beck and Neumann (2011) proposed a model to represent the neurophysiological findings of Majaj et al. (2007), which had explored the temporal and spatial limits of pattern motion selectivity in MT neurons. The MT neurons in their model have less ambiguous motion information compared to V1 neurons, owing to their larger RFs. Feedback connections from MT to V1 gradually disambiguate the motion information generated by MT and V1 neurons. The result of their model is that MT neurons respond to the motion of the individual non-overlapping bars that lie entirely within their RFs without integrating the motion outside their RFs. After a temporal delay, these neurons respond to the movement of the crossing junction of two overlapping bars that are inside their receptive field. As the intrinsic terminators of the bars were visible, MT neurons in their model respond to the motion of the individual bars. As a result, motion of the overlapping bars cannot represent the pattern motion in the presence of the intrinsic terminators. Therefore, the model does not fully replicate the responses of MT neurons to plaids presented inside their RFs (Beck and Neumann, 2011).

Perrone and Krauzlis (2008) also proposed a model to explain the results of the neurophysiological experiments by Majaj et al. (2007). Their model shows that the response of the neurons to the component motions of the pseudo-plaids results from the subregions in the receptive fields of the MT neurons. Our model proposes a different view of this neurophysiological experiment; it shows that the strong component response to the pseudo-plaids can result from the absence of intrinsic terminators in these gratings and, when the gratings overlap, the neurons respond to the pattern motion of the stimulus (Perrone, 2004; Perrone and Krauzlis, 2008).

The temporal dynamics of the pattern motion selectivity of MT neurons in our model accord with neurophysiological results (Smith et al., 2005, 2009). These experiments showed that there is a temporal delay of 50–70 ms in the pattern motion detection of MT neurons, which could be explained by the time required for the propagation of motion information from extrinsic terminators to the other regions of the neural network. However, the responses of component MT neurons do not evolve over time, as is also the case in our model (Smith et al., 2009).

The pattern or component selectivity of MT neurons in the model depends on the strength of the connections from V1-ECRF neurons: the suppressive effect on the signals arising from the extrinsic terminators determine the level of pattern selectivity of MT neurons. Strong connections from V1-ECRF neurons suppress the motion signals arising from the extrinsic terminators and result in component selectivity of the MT neurons. A weak connection from V1-ECRF neurons leads to the dominance of the motion signals generated by cells with RFs located at the extrinsic terminators (in the absence of the intrinsic terminators). Therefore, MT neurons respond to the pattern motion of the plaid as the motion information propagates from extrinsic terminators to other regions. As the levels of pattern selectivity of MT neurons are determined by V1-ECRF neurons and the activity of the V1-ECRF neurons depend on the contrast level of the stimulus, the contrast of the stimulus greatly influences the pattern selectivity of the MT neurons. There is neurophysiological evidence of the effect of contrast on the pattern selectivity of MT neurons. For example, Kumbhani et al. (2008) showed that the pattern selectivity of MT neurons is highly dependent on the contrast of the stimulus gratings: pattern selectivity decreases significantly at lower contrasts. However, component selectivity is contrast invariant in MT neurons. The experiments also show that a decrease in the contrast level of one of the gratings results in a shift of the response of the pattern selective MT neurons toward selectivity for the direction of the movement of the grating with the highest contrast. These findings accord with the expectations of our model, which readily explains why the pattern selectivity of MT neurons depends on the contrast of the stimulus. Our model shows that the pattern selectivity of MT neurons depends on the strength of the connections from contrast sensitive ECR-V1 neurons. As a result, the pattern selectivity of MT neurons depends on the contrast of the stimulus. The model presented by Perrone (2004) does not show that the pattern motion selectivity level of the MT neurons is dependent on the contrast of the crossing junctions or the contrast of the gratings.

The results of our model also accord with neurophysiological findings on the responses of V1 neurons to pattern motion. As the activities of V1 neurons do not evolve over time and, consequently, there is no propagation from the terminators to other regions in V1, the neurons do not detect the pattern motion of the stimulus, which requires the propagation of activity from the extrinsic terminators (Hubel and Wiesel, 1965; Pack and Born, 2001; Pack et al., 2003). Snowden et al. (1991) showed that MT neurons do not respond to pattern motion of transparent random sets of dots. This could be also explained by our hypothesis relating to the importance of the extrinsic terminators on pattern motion selectivity in MT. Since there are no extrinsic terminators formed when two overlapping sets of random dots are presented, MT neurons do not integrate the component motions to respond to the pattern direction.

In summary, the results of our model suggest that the process of pattern motion detection by MT neurons is not necessarily based on the integration of the component motions of the gratings. The key element that determines the pattern or component motion selectivity of the MT neurons is the level of the input received from V1-ECRF neurons, which controls the effect of the extrinsic terminators on the perceived global motion of the stimuli. Therefore, the detection of the pattern motion requires the existence of the extrinsic terminators formed by the overlap of the gratings. The segregation of the different gratings in the RFs of the neurons and the component motion selectivity of the MT neurons to two distinct non-overlapping gratings can also be explained by the model of MT neurons (Majaj et al., 2007). Our model suggests that the segregation of motion signals generated by different objects is the result of the interactions of the surround and the border information received from V1 neurons. This interaction prevents the integration of motion information from different objects. In the case that both gratings are in the same RF, interactions with neighboring MT neurons that have overlapping RFs signify the presence of at least one of the gratings outside of their RFs. This assists MT neurons to differentiate two different moving objects and prevents the global integration of motion signals from different objects.

In future work, it would be advantageous to study the role of extrinsic terminators and center-surround V1 neurons on the pattern motion selectivity of MT neurons by recording from area MT in primates. One method that may be used to examine our hypothesis on pattern motion computation is to examine the temporal dynamics of pattern motion detection for cases of different distances between intersections of the plaids. According to our hypothesis, pattern motion detection results from the propagation of motion information from the intersections of the plaids. If this hypothesis is true, there would be a longer time delay for longer distances between the intersections of the plaids. There are also grounds to extend the model to cover the speed selectivity of the MT neurons and their ability to respond to other types of stimuli by including a model of attention and neurons in MST and V4 areas.

## Data Availability

No datasets were generated or analyzed for this study.

## Author Contributions

Conceptualization: PZ, TK, MI, AB, and DG. Formal analysis: PZ. Funding acquisition: TK, MI, AB, and DG. Investigation: PZ. Methodology: PZ, TK, MI, AB, and DG. Project administration: DG. Resources: DG. Software: PZ. Supervision: TK, MI, AB, and DG. Validation: PZ, TK, MI, AB, and DG. Visualization: PZ. Writing—original draft: PZ. Writing—review and editing: PZ, TK, MI, AB, and DG.

### Conflict of Interest Statement

The authors declare that the research was conducted in the absence of any commercial or financial relationships that could be construed as a potential conflict of interest.

## References

[B1] Adelson E. H. Bergen J. R. (1985). Spatiotemporal energy models for the perception of motion. J. Optic. Soc. Am. A 2, 284–299. 10.1364/JOSAA.2.000284 3973762

[B2] Albright T. D. (1984). Direction and orientation selectivity of neurons in visual area MT of the macaque. J. Neurophysiol. 52, 1106–1130. 10.1152/jn.1984.52.6.1106 6520628

[B3] Beck C. Neumann H. (2011). Combining feature selection and integration-a neural model for MT motion selectivity. PLoS ONE 6:e21254. 10.1371/journal.pone.0021254 21814543 PMC3140976

[B4] Berzhanskaya J. Grossberg S. Mingolla E. (2007). Laminar cortical dynamics of visual form and motion interactions during coherent object motion perception. Spat. Vis. 20, 337–395. 10.1163/156856807780919000 17594799

[B5] Blake A. Troscianko T. (eds.). (1990). Artificial Intelligence and the Eye. Hoboken, NJ: Wiley-Blackwell.

[B6] Born R. T. Bradley D. C. (2005). Structure and function of visual area MT. Annu. Rev. Neurosci. 28, 157–189. 10.1146/annurev.neuro.26.041002.131052 16022593

[B7] Bradley D. C. Goyal M. S. (2008). Velocity computation in the primate visual system. Nat. Rev. Neurosci. 9, 686–695. 10.1038/nrn2472 19143050 PMC4750498

[B8] Dubner R. Zeki S. (1971). Response properites and receptive fields of cells in an anatomically defined region of the superior temporal sulcus in the monkey. Brain Res. 35, 528–532. 10.1016/0006-8993(71)90494-X 5002708

[B9] Grossberg S. (1994). 3-D vision and figure-ground separation by visual cortex. Percept. Psychophys. 55, 48–121. 10.3758/BF03206880 8036093

[B10] Grossberg S. (2014). How visual illusions illuminate complementary brain processes: illusory depth from brightness and apparent motion of illusory contours. Front. Hum. Neurosci. 8:854. 10.3389/fnhum.2014.00854 25389399 PMC4211395

[B11] Grossberg S. (2015). Cortical dynamics of figure-ground separation in response to 2D pictures and 3D scenes: how V2 combines border ownership, stereoscopic cues, and gestalt grouping rules. Front. Psychol. 6:2054. 10.3389/fpsyg.2015.02054 26858665 PMC4726768

[B12] Huang X. Albright T. D. Stoner G. R. (2007). Adaptive surround modulation in cortical area MT. Neuron 53, 761–770. 10.1016/j.neuron.2007.01.032 17329214 PMC1866303

[B13] Huang X. Albright T. D. Stoner G. R. (2008). Stimulus dependency and mechanisms of surround modulation in cortical area MT. J. Neurosci. 28, 13889–13906. 10.1523/JNEUROSCI.1946-08.2008 19091978 PMC2666975

[B14] Hubel D. H. Wiesel T. N. (1965). Receptive fields and functional architecture in two non-striate visual areas (18 and 19) of the Cat. J. Neurophysiol. 28, 229–289. 10.1152/jn.1965.28.2.229 14283058

[B15] Kumbhani R. Saber G. Majaj N. Tailby C. Movshon J. (2008). Contrast affects pattern direction selectivity in macaque MT neurons, in SFN Annual Meeting (Washington, DC).

[B16] Kumbhani R. D. El-Shamayleh Y. Movshon J. A. (2015). Temporal and spatial limits of pattern motion sensitivity in macaque MT neurons. J. Neurophysiol. 113, 1977–1988. 10.1152/jn.00597.2014 25540222 PMC4416600

[B17] Li B. Chen Y. Li B. Wang L. Diao Y. (2001). Pattern and component motion selectivity in cortical area PMLS of the cat. Eur. J. Neurosci. 14, 690–700. 10.1046/j.0953-816x.2001.01689.x 11556893

[B18] Majaj N. J. Carandini M. Movshon J. A. (2007). Motion integration by neurons in macaque MT is local, not global. J. Neurosci. 27, 366–370. 10.1523/JNEUROSCI.3183-06.2007 17215397 PMC3039841

[B19] Movshon J. A. Adelson E. H. Gizzi M. S. Newsome W. T. (1992). The analysis of moving visual patterns, in Book/Report/Conference Proceeding. Frontiers in Cognitive Neuroscience, eds Kosslyn S. M. Andersen R. A. (Cambridge, MA: MIT Press), 148.

[B20] Pack C. C. Born R. T. (2001). Temporal dynamics of a neural solution to the aperture problem in visual area MT of macaque brain. Nature 409, 1040–1042. 10.1038/35059085 11234012

[B21] Pack C. C. Livingstone M. S. Duffy K. R. Born R. T. (2003). End-stopping and the aperture problem: two-dimensional motion signals in macaque V1. Neuron 39, 671–680. 10.1016/S0896-6273(03)00439-2 12925280

[B22] Perrone J. A. (2004). A visual motion sensor based on the properties of V1 and MT neurons. Vis. Res. 44, 1733–1755. 10.1016/j.visres.2004.03.003 15135991

[B23] Perrone J. A. Krauzlis R. J. (2008). Spatial integration by MT pattern neurons: a closer look at pattern-to-component effects and the role of speed tuning. J. Vis. 8, 1–14. 10.1167/8.9.1 18831637

[B24] Rodman H. Albright T. (1989). Single-unit analysis of pattern-motion selective properties in the middle temporal visual area (MT). Exp. Brain Res. 75, 53–64. 10.1007/BF00248530 2707356

[B25] Rust N. C. Mante V. Simoncelli E. P. Movshon J. A. (2006). How MT cells analyze the motion of visual patterns. Nat. Neurosci. 9, 1421–1431. 10.1038/nn1786 17041595

[B26] Salzman C. D. Britten K. H. Newsome W. T. (1990). Cortical microstimulation influences perceptual judgements of motion direction. Nature 346:174. 10.1038/346174a0 2366872

[B27] Smith M. A. Majaj N. Movshon J. A. (2009). Dynamics of pattern motion computation, in Dynamics of Visual Motion Processing (Boston, MA: Springer), 55–72.

[B28] Smith M. A. Majaj N. J. Movshon J. A. (2005). Dynamics of motion signaling by neurons in macaque area MT. Nat. Neurosci. 8, 220–228. 10.1038/nn1382 15657600

[B29] Snowden R. J. Treue S. Erickson R. G. Andersen R. A. (1991). The response of area MT and V1 neurons to transparent motion. J. Neurosci. 11, 2768–2785. 1880548 10.1523/JNEUROSCI.11-09-02768.1991 PMC6575251

[B30] Stoner G. R. Albright T. D. (1992). Neural correlates of perceptual motion coherence. Nature 358, 412–414. 10.1038/358412a0 1641024

[B31] Thiele A. (2007). Reconstructing the world: switching from segmentation to integration allows neurons in area MT to make “sense” of the visual scene. Neuron 53, 623–625. 10.1016/j.neuron.2007.02.008 17329202

[B32] Tsui J. M. Hunter J. N. Born R. T. Pack C. C. (2010). The role of V1 surround suppression in MT motion integration. J. Neurophysiol. 103, 3123–3138. 10.1152/jn.00654.2009 20457860 PMC2888240

[B33] van Santen J. P. Sperling G. (1985). Elaborated Reichardt detectors. J. Optic. Soc. Am. A 2, 300–321. 10.1364/JOSAA.2.000300 3973763

[B34] Zarei Eskikand P. Kameneva T. Ibbotson M. R. Burkitt A. N. Grayden D. B. (2016). A possible role for end-stopped V1 neurons in the perception of motion: a computational model. PLoS ONE 11:e0164813. 10.1371/journal.pone.0164813 27741307 PMC5065146

[B35] Zarei Eskikand P. Kameneva T. Ibbotson M. R. Burkitt A. N. Grayden D. B. (2018). A biologically-based computational model of visual cortex that overcomes the X-junction illusion. Neural Netw. 102, 10–20. 10.1016/j.neunet.2018.02.008 29510263

